# Temperature Resistant Fiber Bragg Gratings for On-Line and Structural Health Monitoring of the Next-Generation of Nuclear Reactors

**DOI:** 10.3390/s18061791

**Published:** 2018-06-02

**Authors:** Guillaume Laffont, Romain Cotillard, Nicolas Roussel, Rudy Desmarchelier, Stéphane Rougeault

**Affiliations:** CEA, List, F-91191 Gif-sur-Yvette CEDEX, France; romain.cotillard@cea.fr (R.C.); nicolas.roussel@cea.fr (N.R.); rudy.desmarchelier@cea.fr (R.D.); stephane.rougeault@cea.fr (S.R.)

**Keywords:** instrumentation, optical fiber, sensing, fiber Bragg grating, multiplexing, temperature, strain, nuclear

## Abstract

The harsh environment associated with the next generation of nuclear reactors is a great challenge facing all new sensing technologies to be deployed for on-line monitoring purposes and for the implantation of SHM methods. Sensors able to resist sustained periods at very high temperatures continuously as is the case within sodium-cooled fast reactors require specific developments and evaluations. Among the diversity of optical fiber sensing technologies, temperature resistant fiber Bragg gratings are increasingly being considered for the instrumentation of future nuclear power plants, especially for components exposed to high temperature and high radiation levels. Research programs are supporting the developments of optical fiber sensors under mixed high temperature and radiative environments leading to significant increase in term of maturity. This paper details the development of temperature-resistant wavelength-multiplexed fiber Bragg gratings for temperature and strain measurements and their characterization for on-line monitoring into the liquid sodium used as a coolant for the next generation of fast reactors.

## 1. Introduction

The advent of high temperature resistant fiber Bragg gratings (FBGs) able to operate in extreme environments for sensing purposes has revitalized research on FBG transducers [1,2,3]. Intense studies are dedicated to the development of FBG sensors capable of continuously withstanding not only temperatures in excess of 600 °C (up to 800 °C and even beyond 1000 °C) but also strain and radiation. These operating conditions are those typically encountered in the next generation of nuclear reactors.

The so-called Generation IV nuclear reactors based on the concept of sodium-cooled fast reactors (SFRs) are being studied at the French Alternative Energies and Atomic Energy Commission (CEA) as part of the six nuclear systems identified by the Generation IV International Forum.

While current nuclear reactors rely on the use of fuel beds of enriched uranium-238, fast reactors keeps the neutrons generated within the core at high speeds. These “fast” neutrons may transform non-fissile materials into fissile ones, consequently broadening the scope of nuclear fuel options and significantly reducing the volume and lifetime of nuclear waste stored within the reactor. The use of unmoderated neutrons to sustain the fission reaction and of a coolant operating at high temperature (typically around 600 °C for liquid sodium) leads to an increase of the expected energy yield within SFR reactors. These improvements come however with rather new and extreme operating conditions. Reaching an availability factor of 80% and a reactor lifetime of 60 years create challenging constraints for on-line monitoring and structural health monitoring (SHM) methods, but also an opportunity to propose and develop innovative instrumentations and sensors [4,5].

The use of “hot” sodium as a coolant to control the temperature of the fission reaction within fast reactors and thus to prevent damage to the core, has triggered the development of innovative sensing solutions able to operate in such a severe and specific environment [6]. Considering that: (i) sodium coolant is opaque; (ii) sodium needs to be operated at a nominal temperature of 580 °C; (iii) this temperature may reach 700 °C in the case of a severe accident situation and not decrease below 97.72 °C (sodium’s melting temperature), any in situ sensing techniques have to be designed in order to withstand high temperatures over an extended period of time without any maintenance and to be sodium compatible. Several innovative solutions have been considered in order to provide tools for on-line monitoring of fast reactor nuclear cores. These are not limited to acoustic and ultrasonic sensors, inspection robots, fission chambers, laser-based spectroscopy techniques for pile gas composition analysis, radiation-hardened thermocouples [6] but also encompass optical fiber sensors [7,8,9,10]. Sensors probing the nuclear core from outside the pool are ideal (ultrasonic sensors) but not always possible. Especially temperature has to be precisely monitored within sodium in order to immediately detect any temperature increase that may be the precursor to a severe accident due to blockage of the sodium coolant along the metal-cladded fuel rods. Variations as low as 10 °C have to be detected in less than one second to prevent the expansion of the blockage to adjacent fuel claddings that could lead to a severe accident.

Regenerated FBGs (R-FBGs) and femtosecond-written FBGs using infrared laser (IR-fs-FBGs) are technological breakthroughs for high temperature sensing and open the way for applications in severe and demanding environments such as those encountered within the nuclear industry [11,12]. Instrumentation of SFR reactors is a typical example. In that case, the needs expressed by the end-users consist basically in the following: (i) to withstand an operating temperature of 580 °C continuously for several years (ultimately during the lifetime of a reactor, four to six decades); (ii) to be radiation tolerant, especially versus high energy neutron flux (fast neutrons, E > 1 MeV, cumulated fluence up to 10^23^ n/cm^2^) but also versus gamma radiations (maximum dose rate up to 30 kGy/h leading to more than 1 GGy over 5 years); (iii) to be compatible with “hot” liquid sodium used as the coolant within the core; (iv) to provide a precision equivalent to that of thermocouples; (v) to analyze hundreds of sensing points and (vi) to have short response time (<500 ms) to detect early coolant blockages leading to potential fuel subassembly meltdown. The ultimate goal for OFS developments for that application would be to obtain a family of qualified FBG sensors withstanding these extreme operating parameters while performing both thermal mapping of nuclear structures but also strain measurements, either in the static regime but also in the acoustic regime in order to propose FBG-based acoustic sensing instrumentations for the implantation of SHM methods (damage detection and localization) in nuclear reactors.

In this paper, we present the manufacturing of: (i) regenerated FBGs using an electrical furnace but also an all-optical process and of (ii) femtosecond laser-written point-by-point femtosecond FBGs using infrared light (IR-fs-FBGs) through the polyimide coating of pure silica core single-mode optical fibers. The presented techniques may not only speed up the manufacturing processes but also improve the mechanical reliability of the FBGs as shown in [13] for phase mask written femtosecond FBGs. Then the temperature stability of both kinds of FBGs with regard to their reflectivity is assessed highlighting their resistance to thermal erasure. R-FBGs and IR-fs-FBGs are appropriate candidates for on-line thermometry even in severe environments such as those encountered in future sodium-cooled fast reactors. Strain sensing is under development using IR-fs-FBGs for operation beyond 400 °C. This paper also shows the test of such fiber Bragg gratings into “hot” sodium in order to confirm the reliability of their packaging for temperature sensing and to determine their response time which is a crucial parameter to guarantee the early detection of abnormal operation of a nuclear core.

## 2. Key Aspects of Fiber Bragg Grating Sensing Technology for Nuclear Applications

### 2.1. Fiber Bragg Gratings Temperature and Strain Transducers

**Figure 1:**
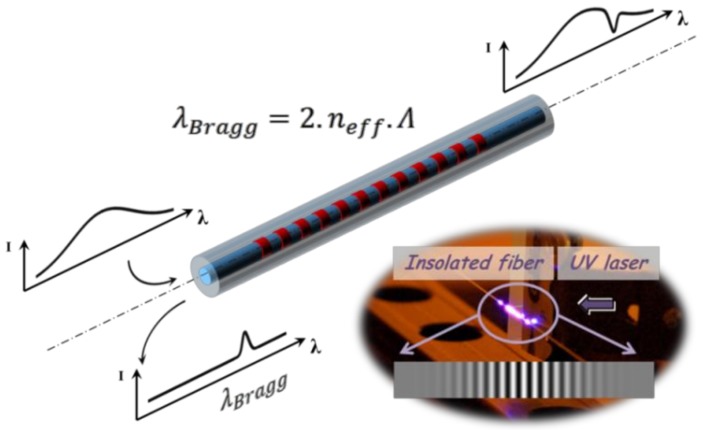
Spectral response of a standard FBG photowritten in a single mode optical fiber together with a picture showing a portion of fiber inscribed using a periodic interference pattern in UV laser light. The observed light at the insolation point stands for luminescence in the blue spectral window.

Fiber Bragg gratings have existed since the photosensitivity phenomenon in optical fibers was discovered in 1978 [14,15]. They are now well-characterized and are used in a variety of high-performance communication and sensing devices. In the field of sensing, standard (type I) FBGs are usually employed to detect physical and/or chemical parameters such as temperature, strain, pressure and refractive index [16,17]. A fiber Bragg grating is a periodic or aperiodic perturbation of the effective refractive index of the guiding core of an optical waveguide, such as single-mode optical fibers. This modulation is obtained through the exposure of the photosensitive fiber’s core to UV laser light. A fiber Bragg grating reflects a narrow spectral band located at the Bragg wavelength (see Figure 1) according to the following relationship between n_eff_ the fundamental mode refractive index and Λ the grating’s pitch:

$$\lambda_{\mathrm{Bragg}}=2\times n_{\mathrm{eff}}\times \Lambda$$

By simply changing the grating’s pitch, one can easily change the Bragg wavelength from one FBG to another written at a different location along the same optical fiber. This is the basic principle of spectral multiplexing applied to FBG transducer. Each FBG sensor is identified by its Bragg wavelength. Any change of the measurand (temperature, strain and/or pressure for instance) observed by a given FBG induces a spectral shift of its Bragg wavelength. The measurand is spectrally encoded in the form of a spectral shift while the sensor identity is given by the absolute Bragg wavelength. Taking advantage of the low attenuation of single mode optical fiber in the near infra-red spectral window, FBGs are usually photowritten in order to get a Bragg wavelength in the 1.55 µm window.

Each FBG transducer is identified through its own Bragg wavelength which is user-defined at the photowriting step thanks to accurate control of the grating’s pitch. Both the spatial position of each FBG on a single optical fiber and the spectral window containing its Bragg peak are known at the sensing line fabrication step. The Bragg wavelength not only identifies the transducer but it also contains the measurand information: the parameter to be measured by each FBG transducer is spectrally encoded in the form of a Bragg wavelength shift. Using a calibration protocol, the absolute value of any measurand is retrieved from the Bragg wavelength shift and from the corresponding transducer’s sensitivity, for instance versus temperature.

Any change of the temperature of a FBG transducer induces a spectral shift of its Bragg wavelength. This phenomenon is related mainly to the thermo-optic effect but also to the glass dilatation. At 1550 nm and over a limited range of temperature (let say 0 °C to 200 °C), the thermal sensitivity of a FBG temperature sensor is roughly constant and equals to [17]:

$$\frac{\Delta \lambda_{\mathrm{Bragg}}}{\Delta T}\left[1550\;\mathrm{nm}\right]\Delta 11.3\;\mathrm{pm}/{}^{\circ}C$$

Over a broader range of temperature, the thermo-optic coefficient of silica changes non-linearly with temperature. Thus it is necessary to calibrate the sensor using for instance a polynomial fitting curve in order to keep precision to several tenths of a degree Celsius.

With regard to strain, a FBG transducer experiences a low-wavelength side (blue-shift) or a long-wavelength side (red-shift) shift depending on whether a tensile or a compressive strain is applied to the optical fiber. At 1550 nm and at room temperature, the strain sensitivity of a FBG is roughly constant and equals to [17]:

$$\frac{\Delta \lambda_{\mathrm{Bragg}}}{\Delta \varepsilon }\left[1550\;\mathrm{nm}\right]\Delta 1.2\;\mathrm{pm}/\text{µ}\varepsilon$$

### 2.2. FBG Wavelength Multiplexing

**Figure 2:**
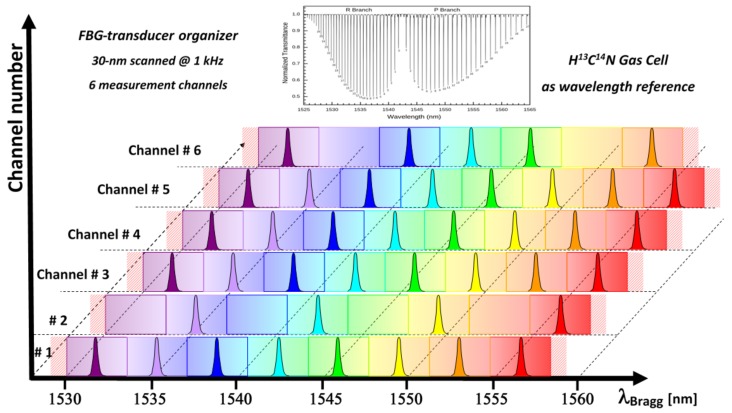
FBG transducers typical spectral allocation table to prevent spectral crossing. The sensor number is indicative: several tens of sensors may be easily interrogated depending on the measurand dynamic. Also shown at the top: a wavelength reference channel based on a gas cell providing absolute spectral calibration in real time of the monitoring system.

As the Bragg wavelengths allow transducers identification in the spectral domain, one can easily multiplex several FBG transducers on a single optical fiber, but care has to be taken in order to prevent spectral crossing of adjacent FBGs: thus end users allocate a specific spectral window to each FBG based on the *a priori* knowledge of its expected wavelength shift. Figure 2 shows an example of such a spectral allocation table for FBG sensors dispatch over six different optical fiber channels over the C-band telecom window. As a spectral reference, the transmission spectrum of an acetylene gas cell is also shown at the top of Figure 2. Such a spectral reference may be integrated into wavelength scanning systems based on tunable laser or scanning Fabry-Perot to get accurate Bragg wavelength measurements [18]. The maximum number of FBG sensors depends not only on the instrumentation used to interrogate the sensors, but also on the expected wavelength shift and on the gradient between adjacent sensors. Higher sensor densities can be reached in the case of low gradient situations, for instance. Thus, typical FBG monitoring systems based on optical spectrum measurements may interrogate from several up to tens of sensors depending on the application. Other interrogation techniques such as those using both wavelength and time division multiplexing schemes or those based on quasi-continuous gratings and optical frequency domain reflectometry [19] can address thousands of sensing elements but with tradeoff in term of measuring range and speed, but also in term of stand-off length for remote operation.

**Figure 3:**
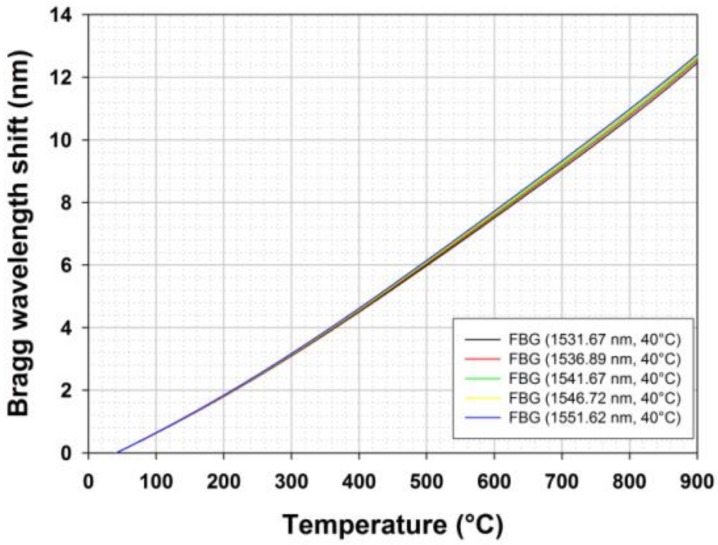
Bragg wavelength shift with temperature up to 900 °C for five regenerated FBGs realized and calibrated at CEA List laboratory. The seed FBGs were written using a 244 nm continuous-wave Argon Ion laser (Fred Innova 300C from Coherent) into H_2_-loaded smf-28 optical fibre. Regeneration was performed at 910 °C in a tubular furnace.

For instance, in case of a temperature sensor dedicated to measure temperature changes from 0 °C to 600 °C as could be expected in the case of SFR nuclear core monitoring, the maximum wavelength shift is expected to be roughly 8 nm and 13 nm over a 900 °C wide range. Figure 3 shows a typical calibration curve obtained for five regenerated FBGs studied from 40 °C up to 900 °C. These curves have been obtained using a tubular furnace with three heating zones (model TZF 12/38/850/3216, Carbolite, Eragny-sur-Oise, France 12/38/850 heating up to 1200 °C and with a tube whose length and inner diameter are respectively 850 mm and 38 mm) The FBGs data have been acquired using a flattened ASE optical source and a spectrometer (MS9710C, Anritsu, Villebon-sur-Yvette, USA, France, resolution of 0.05 nm). The temperature is obtained using a HP34970A data logger (Keysight Technologies, LEs Ulis, France) and a bundle of five type N thermocouples (purchased from Correge, Chaignes, France) distributed along the length of the tubular furnace. The thermocouples’ accuracy is given by the manufacturer as ±1.5 °C from room temperature to +375 °C and as ±0.004*T between 375 °C and 1000 °C, so the temperature accuracy is on the order of ±2.8 °C at 700 °C. Thermocouple data are used to get the temperature profile along the furnace at each time and thus to extrapolate the temperature at the location of each FBG under calibration. The non-linear behavior of the Bragg wavelength with temperature is clearly visible while the evolution of the thermo-optic coefficient with the Bragg wavelength explains the difference between each FBG.

### 2.3. FBGs and Radiative Environments

There is a wide diversity of scientific and industrial applications for FBG sensing technology [2,12,16]. FBG sensors are not limited to research and laboratories testing: they are now widely used in many industrial fields and have gain in maturity through two decades of field tests by numerous international teams. Starting from the basic parameters or measurands that could be addressed by a given FBG transducer (temperature, strain, pressure and refractive index), one can also measure a diversity of physical and chemical parameters thanks to appropriate functionalization or packaging. Thus a non-exhaustive list of available FBG-based sensor comprises [15,20]: temperature sensor, strain gauge, pressure sensor (static and dynamic), extensometer, inclinometer, mono and/or multi-axes accelerometer, refractometer [21], and chemical or biochemical agent detection [22,23,24]. This diversity of addressable measurands together with the excellent metrological properties of FBG sensors explain the corresponding diversity of industrial application fields: civil engineering, materials, composite materials, railway and sea transport, aeronautic, space, chemistry and biology, defense, and nuclear energy. Being one of the most demanding application fields for FBG sensing technology, nuclear energy applications and especially the instrumentation of future nuclear reactor concepts with improved safety and security represent a great challenge for sensor developers, triggering innovations even around the well-known concept of fiber Bragg grating transducers and addressing specific issues such as the impact of radiation.

Beyond the scope of this paper, the behavior of fiber Bragg gratings under radiation has to be specifically addressed for nuclear applications (nuclear power plants, nuclear cores) but also for fundamental research (fusion studies using Tokamaks, accelerators used in particle physics). Similar studies but at lower dose are also mandatory for space applications (satellite or launcher instrumentation). Irradiation of optical fibers and fiber Bragg grating transducers have strong influence on any measurement due to radiation induced attenuation (RIA), radiation induced emission (RIE) and radiation induced compaction (RIC) [25,26,27,28]. RIA leads mainly to a reduction of the signal-to-noise ratio for a given interrogation signal while RIA and RIC (compaction of the silica glass matrix in case of irradiation reaching high fast neutron fluence) are both responsible for drifts of the Bragg wavelengths, and thus to increasing measurement errors. Generating a spectrally-wide emission into the optical fiber, RIE has usually no impact on FBG measurements due to the spectral finesse of the Bragg peaks (typically hundreds of picometer for the Bragg peak’s FWHM) depending on the grating’s length [26].

FBGs behavior under radiation is also a function of the optical fiber itself (glass, dopants, refractive index profile, protective coating, manufacturing process) and of the thermal history (thermal annealing procedure) experienced by the grating. The influence of radiation on FGBs is further complicated by the diversity of grating types (type I, II, regenerated, micro-voids etc…) obtained with the various inscription processes and lasers (various UV-wavelengths making use of the fiber’s photosensitivity, femtosecond lasers for type II and microvoid gratings etc…). Rarely characterized due to the lack of suited experimental platforms (one has to plan directly experiments into the few research reactors available worldwide) is the influence of high temperature irradiation of FBGs. Let us mention that high temperature may lead to partial recovery of irradiated components but once again on-line experiments are required as dynamic behavior of FBGs under harsh environments mixing high temperature and radiation (gamma, thermal and fast neutron) is hard to anticipate and predict [26,27,28,29]. Such studies are mandatory to determine the metrological performances (resolution, accuracy, measuring range, dynamic) of any FBG-based “nuclearized” sensor but also their realistic lifetime.

Preliminary experiments have been done at CEA List on regenerated FBGs exposed to X-ray radiation coming from a high energy linear accelerator having a 9 MeV nominal energy. This irradiator was the only one available at CEA at the time of the experiment and able to accept (regarding both the safety rules and the experimental area) a high temperature furnace within the irradiation area. A tubular furnace has been positioned in contact to the exit window of the X-ray generator while taking care to avoid any overheating. The regenerated FBGs were photowritten in a SMT-A1310H germano-silicate polyimide-coated single-mode optical fiber from OFS Company (Norcross, GA 30071 USA) using a continuous-wave argon laser emitting at 244 nm and a Lloyd mirror interferometer. Spectral measurements are performed using a T100S-HP tunable laser and a CT-400 component tester, both from Yenista (Lannion, France). Preceding the photo-inscription, the polyimide coating is first chemically removed at the desired FBG location before being H_2_-loaded during two weeks at room temperature. This eliminates the possible influence of the coating on the Bragg wavelength shift of the gratings. The regeneration process is performed after the photo-writing by raising the temperature of the grating close to 900 °C long enough to ensure complete regeneration. A first regenerated FBG is used for the irradiation experiment at room temperature while a second regenerated FBG served as a reference for the tests conducted at high temperature. The heated regenerated FBG is centered inside a tubular furnace placed perpendicular to the direction of propagation of the radiation beam. For each temperature step, both the furnace temperature and the Bragg wavelength shift are controlled in order to check whether a stable state is reached before starting the irradiation. The effective dose received by the fiber is estimated using the specification of the linear accelerator’s manufacturer stating a dose rate of approximately 28 Gy/min at one meter from the tungsten target, which translates into 92 Gy/min for the FBGs in the furnace and 414 Gy/min for the reference FBG tested at room temperature and put closer to the tungsten target. For each test, the experiment lasted roughly 6 h. The potential dose attenuation caused by the walls of the oven is assumed negligible. The furnace used for this experiment has its electronic controller positioned several meters away from the heating elements and thus outside the radiation beam.

The overall BWS ∆λ_Bragg_ is tracked using a 3rd order polynomial fit on the continuously acquired FBG reflection spectrum. The spectra are acquired using an external cavity tunable laser together with a CT-400 component tester from Yenista (Lannion, France). The oven temperature is monitored with a thermocouple placed close to the FBG and then converted into temperature-induced BWS ∆λ_temperature_ using temperature calibration laws previously obtained for regenerated FBGs processed in the same type of optical fiber:

(1) $$\Delta \lambda_{\mathrm{Bragg}}=\Delta \lambda_{\mathrm{irradiation}}+\Delta \lambda_{\mathrm{temperature}}$$

**Figure 4:**
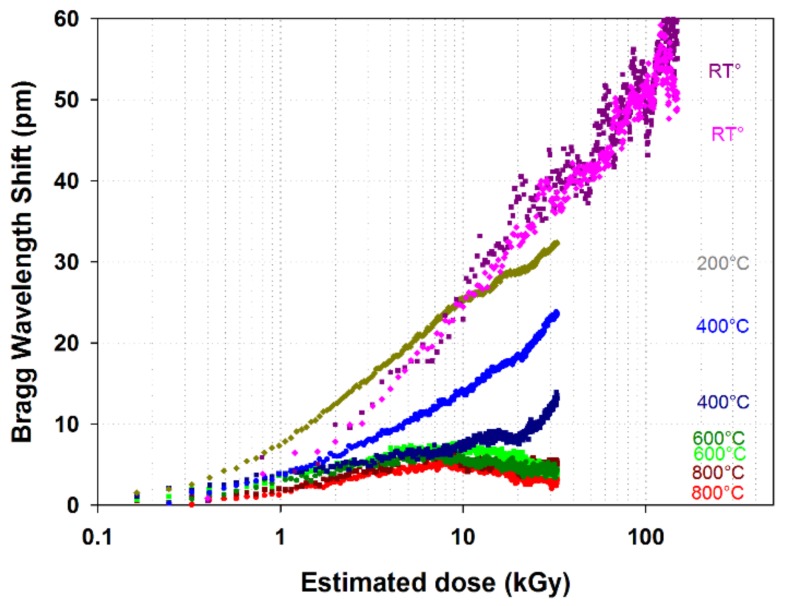
X-ray radiation-induced Bragg wavelength shift measured at different temperatures. The heated regenerated FBG received a total dose of 33kGy after 6 h at an average rate of 92 Gy/min. The reference FBG at room T° [M4] received a dose of 148 kGy at an average rate of 414 Gy/min. The T° compensation data used at 200 °C was partly extrapolated due to a failure in the acquisition. The absence of temperature stabilization and compensation for the curves at room T° explains their higher fluctuation.

Assuming that the thermocouple is not affected by the radiation and that it measures the actual temperature of the FBG, the radiation-induced BWS ∆λ_irradiation_ can be isolated from the temperature shift of the oven with a subtraction according to the above equation. At temperature greater or equal to 400 °C we have also assumed a complete recovery of the irradiated regenerated FBG between two consecutive irradiations in the heating furnace. Therefore, the irradiation performed at temperature lower than 200 °C where this recovery is not verified are performed at the end of the experiments. Results are shown on Figure 4. The radiation-induced BWS is clearly decreasing at higher temperatures. This highlights the recovery capability of the silica substrate during radiation experiments at high temperature. Above 600 °C, the dose rate produced by our installation was too low to observe a significant difference in the radiation-induced BWS behavior. These results are consistent with literature which states an accelerated recovery process of materials at increasing temperatures [30]. Discrepancies in the two sets of data at 400 °C may come from an error in the assumptions that lead to Equation (1) and calls for further experiments to assess the hypothesis made and to check repeatability.

As illustrated in Table 1, the radiation-induced temperature error for regenerated FBG decreases at high temperature mainly due to the material recovery. These preliminary results highlight the interesting behavior of regenerated FBG for temperature measurement in extreme environments combining high temperature and radiation and also the need for in line monitoring of irradiated heated temperature-resistant FBG in order to evaluate their cross-sensitivity to radiation. Such experiments require other experimental facilities than the one used in this preliminary study involving only X-ray generator. Higher dose and dose rate are necessary to exacerbate the effect of the gamma rays on the FBGs especially with regards to ambient temperature changes. Unfortunately very few irradiators are designed both for continuous irradiation in order to reach at least MGy dose levels and simultaneously heating the component under test at several hundreds of degree Celsius during the irradiation. So at CEA List we are preparing experiments into research reactors. Whereas such experiments require the availability of such a facility together with a significant investment in preparation time they will provide insightful data not only for gamma radiations but also with regards to neutron flux and high temperature. Such tests are mandatory to qualify fiber sensors for nuclear needs, both for optical fibers such as in [29] but also for transducers and components such as temperature resistant FBGs.

## 3. Manufacturing Techniques of High Temperature Resistant Fiber Bragg Gratings

Several methods have been proposed in the literature to increase the thermal stability of the standard type I fiber Bragg gratings, usually inscribed into germano-silicate photosensitive optical fibers, especially for sensing applications in harsh environments. In this study, we are focusing on two main techniques recognized as promising and flexible for sensing purposes in harsh environments: the regeneration of “seed” FBGs [2,11] but also the inscription of FBGs using ultrafast lasers, especially the gratings based on “microvoids” [1,31].

### 3.1. Regenerated FBGs

**Figure 5:**
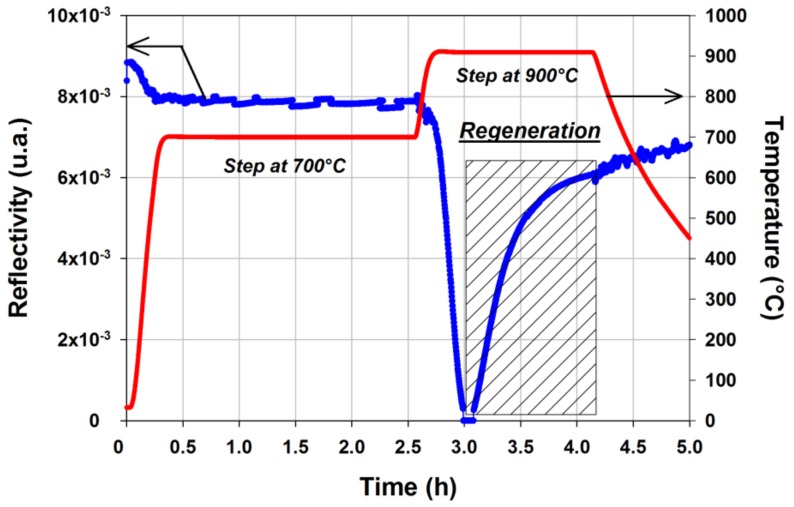
Evolution of the FBG reflectivity versus time during the regeneration process of a seed FBG photowritten into a SMF-28 optical fiber H_2_-loaded during two weeks at room temperature prior to the grating’s inscription with a cw frequency-doubled argon ion laser emitting 100 mW at 244 nm (experiment performed at CEA List).

Regeneration of “seed” FBGs is an attractive approach increasing the operating lifetime of FBGs employed in high temperature environment [2,11]. From a practical point of view, it consists in annealing a standard saturated FBG, called a “seed” grating. This seed FBG is first erased through an annealing at temperature closed to 900 °C and subsequently a new Bragg peak appears on the spectrum. Its reflectivity increases gradually up to a maximum level depending on the annealing protocol, on the seed grating initial reflectivity and on the fiber composition and fabrication process. Stopping the annealing at this step gives rise to a so-called regenerated FBG. Figure 5 shows the typical evolution of the reflectivity during such a regeneration process. Spectral measurements are performed using a flattened C+L ASE broadband source and a MS9710C Anritsu spectrometer. The obtained gratings are stable at temperature up to 900 °C, namely that of the regeneration process. On Figure 5, the regeneration process consists in a first step at 700 °C during two hours followed by the regeneration itself at 900 °C. It has been experimentally observed that a preliminary heating at 700 °C leads to regenerated FBGs with a higher reflectivity for seed grating written in the H_2_-loaded SMF-28 fibre used in this experiment. But this behavior and the regeneration process as a whole are strongly dependent on the optical fibre itself, on its chemical composition, on the diffusion of gases inside the glass such as hydrogen and on the fibre drawing and seed grating photowriting processes [32]. The regeneration protocol can be adjusted with regards to several objectives such as for instance its efficiency but also its duration or the mechanical reliability of the regenerated FBG. Fast regeneration scheme have already been demonstrated either in tubular furnace [33] or using a CO_2_ laser to heat the seed FBG [34].

**Figure 6:**
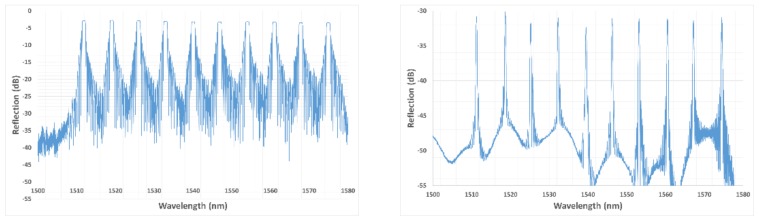
Reflection spectra before and after regeneration for a line of ten wavelength-multiplexed Fiber Bragg Gratings. The regeneration has been realized at CEA List using a three heating zones tubular furnace on an FBG array written in H_2_-loaded smf-28 optical fibre and a KrF laser emitting at 248 nm.

The regeneration process occurring in silica glass is an attractive solution suited to the development of wavelength multiplexed FBG sensing lines (see Figure 6). Regenerated FBGs open the way for many stringent applications and processes involving high temperature operating conditions. They combine the well-known advantages of standard FBG sensors—EM immunity, remote interrogation, spectral multiplexing capabilities, miniature size, reliability, reasonable cost per sensing point—while proposing an increased stability in temperature (up to 900 °C) and preserving the finesse of the Bragg peak. Deployment of regenerated FBG arrays for nuclear sensing applications requires not only the development of efficient regeneration methods suited to the topology of the sensing lines, the use of high dynamic monitoring units to cope with their low reflectivity and collective calibration of the sensing points to propose accurate temperature (or strain) measurements with regards to conventional sensors (thermocouples) but also the testing of their behavior (drift, erasure, radiation induced attenuation) under real conditions combining gamma/neutrons radiation and high temperature (typically beyond 300 °C).

**Figure 7:**
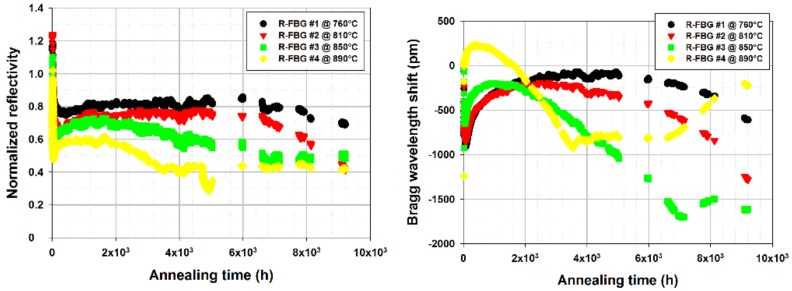
Evolution of the Reflectivities (**right**) and Bragg Wavelength Shifts (**left**) of four regenerated FBGs over a one year-long thermal ageing experiment up to 890 °C, published by the authors in [35].

As shown on Figure 7, long term thermal ageing of arrays of regenerated FBGs have been performed at temperature up to 890 °C. Four regenerated FBGs have been written in a SMT1310H single-mode optical fiber, packaged individually into metallic capillaries and inserted in a vertical tubular furnace. This test has shown that regenerated FBGs are able to withstand a one-year long thermal ageing at temperature very close to their regeneration temperature without disappearing but with moderate wavelength drift that may be attributed to viscosity change of the silica substrate and to packaging issues [35].

### 3.2. All-Optical Setup for FBG Regeneration

**Figure 8:**
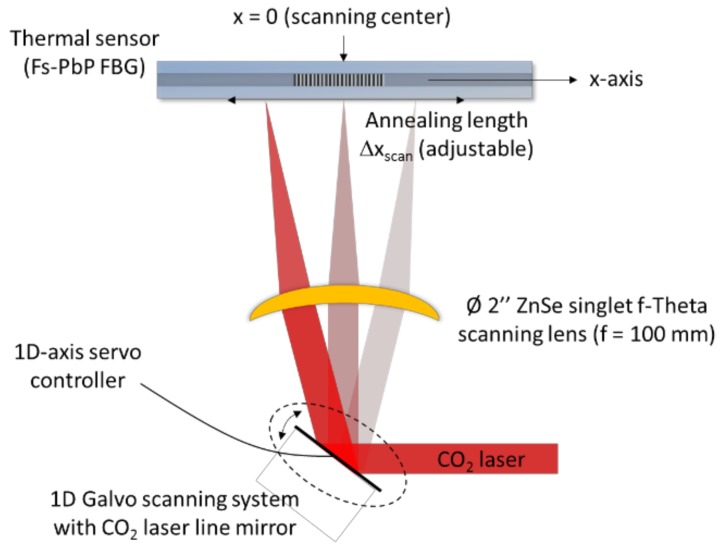
Scheme of CO_2_ laser-based annealing setup to regenerate a FBG.

Regenerated FBGs are usually obtained thanks to a thermal engineering process making use of a standard tubular furnace. This approach presents several limitations, not only in in terms of achievable topology of the multiplexed sensing points along the fiber strand but also in term of mechanical reliability. Indeed a whole strand of fiber (as long as the furnace itself) has to be heated up to roughly 900 °C while only centimeter long segments containing the individual seed FBGs should be heated for the regeneration to occur. The unnecessary heated length of fiber results in cumulated defects and the fiber becomes brittle. Another scheme developed and realized at CEA List is exposed on Figure 8 to perform the regeneration while preserving the mechanical reliability of the gratings: both inscription and regeneration are performed optically as it has been previously published by other teams in the literature [34,36]. While the seed FBG photowriting makes use of a traditional pulsed UV laser operating at 248 nm with nanosecond pulses (KrF laser) and of a phase mask, the regeneration itself is performed using a CO_2_ laser heating only a small length of fiber containing the seed FBG. This all-optical regeneration setup scans the seed FBG with a laser spot using a 1D galvanometric scanner using a gold-coated mirror and an f-theta lens in order to focus the laser beam along the optical fiber axis. Both scan speed optimization and gating approach of the scanner’s driving command are used to get a uniform temperature profile, spatially and temporally, along the length of the FBG. While proposing a solution to heat only the necessary part of the optical fiber, this setup also allows us to get rapid rising time of the temperature along the grating. As a consequence, this all-optical heating setup is also particularly useful to study the response time of temperature-resistant FBG with respect to temperature increase.

**Figure 9:**
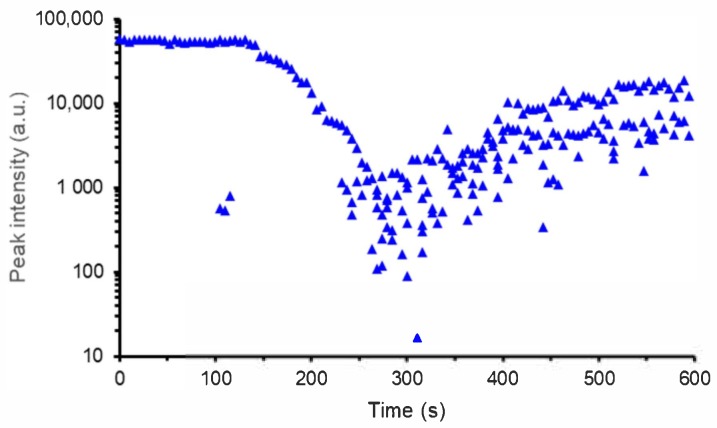
Regeneration of FBG using CO_2_ laser annealing. Annealing parameters: scan movement: sinus + gating signal, f_SCAN_ = 100 Hz, DX_SCAN_ = 14 mm, PWM < 40%, f_CO_2__ = 20 kHz.

The regeneration illustrated on Figure 9 is obtained from a Type-I seed grating inscribed on a SM1500(9/125)P optical fiber from Fibercore (Southampton, UK) and using a KrF laser emitting at 248 nm with a maximal pulse energy of 140 mJ and a maximal repetition rate of 100 Hz. The optical fiber is exposed to the writing beam until saturation. The seed FBG used in this experiment has a length of 5 mm and a reflectivity of 99.986%. The noise observed during the regeneration is mainly due to temperature fluctuations along the annealed FBG coming from air convection around the fiber. This can be alleviated thanks to ZnSe window to isolate the optical fiber while allowing the infrared beam to be transmitted to the fiber.

### 3.3. Femtosecond Fiber Bragg Gratings

**Figure 10:**
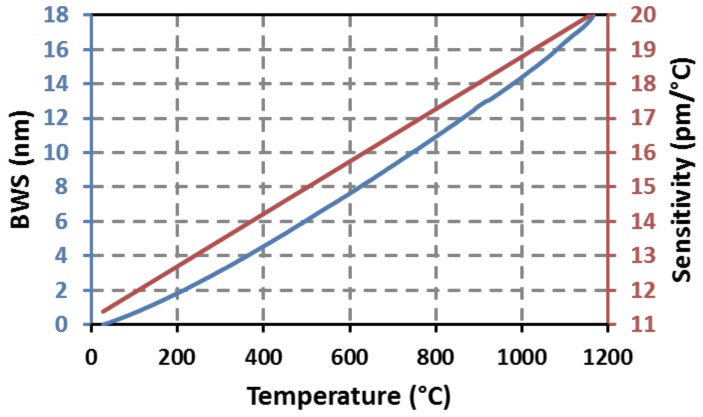
Calibration curve showing the Bragg Wavelength Shift (BWS) of a IR-fs-PbP FBG with respect to temperature and evolution of its thermal sensitivity according to the operating temperature.

To operate beyond the regeneration temperature or to get temperature resistant FBG with a greater mechanical reliability, advanced photowriting techniques based on ultrafast laser are currently developed in order to propose “microvoids” based FBG transducers [1,12]. By focusing intense pulsed laser beams from a femtosecond near-infrared laser, one can generate a micro explosion into the core of the optical fiber that leads to a localized micro void. By simply translating the fiber in front of the focusing optic at a constant speed, an array of micro voids with a constant pitch is obtained along the fiber. This array of micro voids gives rise to a spectral resonance corresponding to a Bragg peak. These femtosecond FBGs require no intrinsic photosensitivity property of the optical fiber. Thus it becomes possible to get FBG whatever the optical fiber’s substrate. This is of particular importance for nuclear applications as pure silica core fibers is one of the most performing fiber platform. Femtosecond writing techniques such as the above point-by-point (PbP) approach is a very promising way to get arrays of temperature stable FBGs. Micro voids-based femtosecond FBGs have been demonstrated to be operational at temperature up to 1200–1300 °C in silica core optical fiber. Beyond 1300 °C sapphire fibers have to be used and type II femtosecond FBGs have already been realized in such substrates. The use of near infrared femtosecond laser presents the inherent advantage of a beam able to write gratings even through the protective coating of the fiber such as polyimide. Even if this coating does not withstand temperature beyond 350 °C, it is particularly useful to get a preserved polymer coating for packaging and deployment issues. Figure 10 shows a typical calibration curve of such an IR-fs-PbP FBG obtained within a standard SMF-28 optical fiber. The IR-fs-PbP FBG has been written at CEA List using a Legend Elite femtosecond laser from Coherent (Santa Clara, CA, USA) , emitting 120 fs pulses (5 mJ/pulse) at a central wavelength of 800 nm and with a repetition rate of 1 kHz. The PbP FBG are obtained by translating the optical fiber with an air-bearing stage (ABL-10100-LN from Aerotech, Pittsburgh, PA, USA) at a constant velocity in front of a non-moving objective whose numerical aperture is 0.65. The calibration curve on Figure 10 is very similar to the one obtained for regenerated FBGs (see Figure 3) as the substrate (Ge-doped smf-28 optical fibre) is the same for both gratings.

**Figure 11:**
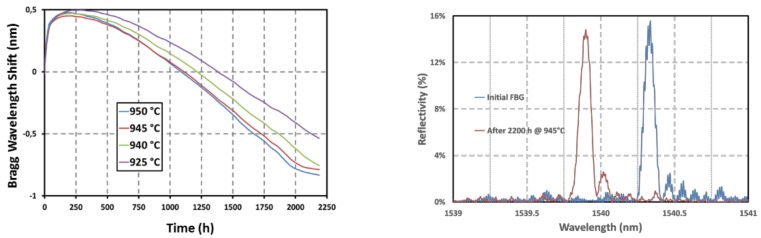
Bragg Wavelength Shift (**left**) of four identical IR-fs-PbP FBGs written and annealed at four different temperatures at CEA List laboratory together with room temperature reflection spectrum (**right**) for one of these FBGs before and after annealing during 2200 h at 945 °C.

As for regenerated FBGs, long term annealing at high temperature of IR-fs-PbP FBGs is mandatory to assess their stability over realistic period of time. Four first order IR-fs-PbP FBGs have been written at CEA List in four pieces of SMT-A1310H germano-silicate polyimide-coated single-mode optical fiber. They have the same Bragg wavelengths (design wavelength of 1540 nm) to avoid any discrepancy that may arise from the dependence of the thermal sensitivity with the wavelength. The writing source is a Ti:Sa femtosecond laser emitting 120 fs pulses at 800 nm and with a repetition rate of 1 kHz. This grating has been packaged in a 1 mm in diameter metallic capillary made of 316L material. It has been tested in a horizontal tubular furnace with three heating zones. The reflection spectrum of each grating has been monitored using an external cavity tunable laser (Tunics T100S-HP from Yenista) and a four channels component tester (CT-400 from Yenista). The ageing experiment lasted 2200 h at four different temperatures: one temperature per grating, each grating carefully positioned at different locations inside the furnace. Using the thermal gradient within the furnace, the ageing temperatures were chosen to be: 925 °C, 940 °C, 945 °C and 950 °C. As shown on Figure 11 for the IR-fs-PbP FBGs tested at 945 °C, its reflectivity, measured once the grating is returned at room temperature, experiences a drop of less than 5% after 2200 h at 940 °C continuously with regard to its initial reflectivity also measured at room temperature. The data for the other IR-fs-PbP FBGs are compiled in Table 2. Two gratings, annealed respectively at 950 °C and 940 °C, show a greater change in reflectivity after the thermal ageing but this is due to the furnace’s gradient in temperature over the length of these gratings leading to a chirp effect and thus to a broadening of their Bragg peaks and a decrease of their reflectivity. This chirp effect is then frozen into the glass once the gratings return to room temperature. The drifts in Bragg wavelengths measured at room temperature before and after annealing correspond to blue shifts of increasing amplitude with regards to the annealing temperature. The IR-fs-PbP FBG annealed at the highest temperature exhibit the largest blue shift reaching 520 pm for a temperature of 950 °C while the grating at 925 °C experiences a blue shift of 180 pm for a temperature of 925 °C. In case of type I FBG written in photosensitive optical fibre, such a drift is usually related to thermally-activated erasure of the mean refractive index change photoinduced along the length of the grating. For the PbP FBGs tested in this study, the blue shift may come from changes in the microvoids pattern but also from changes of the densified shell surrounding each void [37].

This clearly highlights the stability in reflectivity of these gratings. Regarding the Bragg wavelength shift (BWS), Figure 11 shows that there is an initial increase during the first 100 h followed by a decrease by almost 1.3 nm. Then the two hottest FBGs tend to stabilize. Longer tests are still to be performed. The curves showing the BWS versus time for these IR-fs-PbP FBGs are very similar to those obtained for regenerated FBGs (see Figure 7). So, we assume that these drifts may be attributed to the ageing of the optical fiber itself (through refractive index profile and mode confinement changes for instance), to the change of the glass viscosity and/or to the packaging of the gratings. The consistency of these drifts suggests the possibility to predict them for compensation purposes. Other options to limit this drift could be to anneal the gratings prior to their use as sensors and/or to use optical fiber with higher glass transition temperatures.

## 4. Deployment of Temperature-Resistant FBGs into Sodium

Experiments for the evaluation of temperature-resistant FBGs in liquid sodium have been conducted. These tests aim to clarify the compatibility with high temperature (550 °C) liquid sodium of packaged optical fiber temperature probes involving in the presented results regenerated FBGs. The response time of a regenerated FBG-based temperature probe has been characterized together with the ability to measure temperature gradients in a hot glove box compatible with liquid sodium. The presented results primarily reflects the influence of the probe’s packaging rather than the influence of the R-FBG transducer. Of importance is the fabrication material and thickness of the metallic capillary, but also the sealing of the distal end of the probe to prevent ingress of liquid sodium inside the sensing probe and the acquisition rate of the FBG monitoring system to ensure the right time resolution in order to resolve the response time of the probe. The grating itself, here R-FBG transducers, are of importance with regards to the tested temperature, reaching up to 500 °C in sodium. R-FBGs being stable at these temperatures, neither reflectivity drop due to thermal erasure nor wavelength drift due to thermal ageing of the grating may have an influence on the presented results.

**Figure 12:**
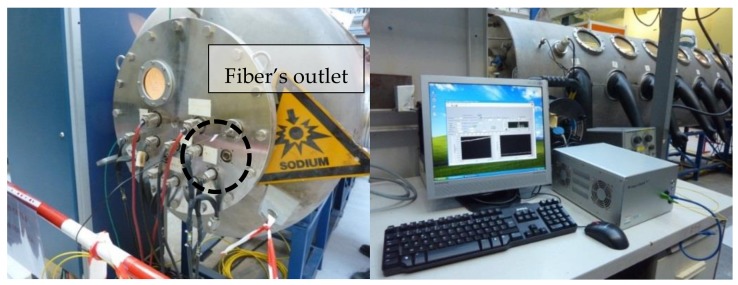
On the left, the CEA/Penelope glove box for testing of sensors in heated liquid sodium and on the right CEA LIST proprietary FBG monitoring system deployed nearby the Penelope facility located at CEA/Cadarache research centre.

All the tests in liquid sodium are conducted in the Penelope glove box operated at CEA Cadarache (see Figure 12). This box allows to safely heat liquid sodium up to 550 °C and thus to realize the tests in immersion of materials, sensors and/or instrumentation prototypes.

For testing, we have realized a sensing line comprising five regenerated FBGs. The gratings are 2 mm in length and spaced by 3 mm. The sensing optical fiber is inserted in a metallic capillary made of Inconel 600. The extremity of the capillary inserted in sodium was sealed. A Swagelok seal fitting is used for the capillary outlet. A proprietary interrogation system is located nearby the Penelope box (see Figure 12) and is used to demultiplex the FBGs at a rate of 1 kHz and a precision of 1 picometer. The system relies on a client/server approach. The opto-electronic unit can be remotely positioned with respect to the sensor (connected to the opto-electronic unit thanks to outdoor optical fiber cables) and its continuous operation controlled thanks to a client software interfaced through an Ethernet link to the unit (thus a remotely installed computer may controlled one or several opto-electronic units positioned in a dedicated cabinet). Thus one can control and/or process measurement data in real time without any impact on the main unit operation.

**Figure 13:**
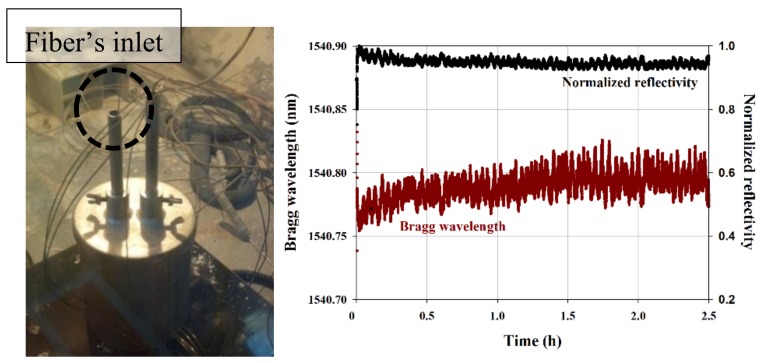
Sodium box installed in the Penelope facility and heated from the bottom (**left**). The fiber’s inlet is highlighted: a single capillary is enough to position 5 multiplexed regenerated FBGs; the other inlet is for three thermocouples. On the right, stability of a regenerated FBG in hot sodium (500 °C).

A specific liquid sodium box was constructed and inserted into the Penelope facility in order to realize measurements in hot sodium and to characterize gradients of temperature. Thus the box is heated from its bottom. The sensors are inserted from the top as shown on Figure 9. Three thermocouples are also immersed in sodium at a close vicinity from the optical sensors. They are assembled all together thanks to a main metallic body also plunged into sodium. Stability of the sensing head during immersion in sodium at 500 °C demonstrates the compatibility of the packaging with liquid sodium. As shown on Figure 13, a single capillary of 1 mm in outer diameter is enough to insert five high temperature resistant FBG sensors and to send the optical signal back to a remote monitoring system. With the same intrusivity, a sensing line with several tens of FBG sensors multiplexed on a single fiber is straightforward to implement. Thus the routing of the FBG temperature probe is easier than the routing of several thermocouples. During the 2.5 h-long immersion in sodium, we do not observe any significant evolution of the Bragg wavelength and of the reflectivity. Initial evolution stands for stabilization of temperature inside the box. The observed ripple is due to the regulation of the heating system. With a typical temperature thermal sensitivity of 15 pm/°C at 500 °C, the observed ripple of ±2.5 pm corresponds to oscillation of ±0.2 °C in temperature. Ripple is also observed on the reflectivity due to the spectral dependence of the optical power density and the lack of normalization for the reflected Bragg peak.

**Figure 14:**
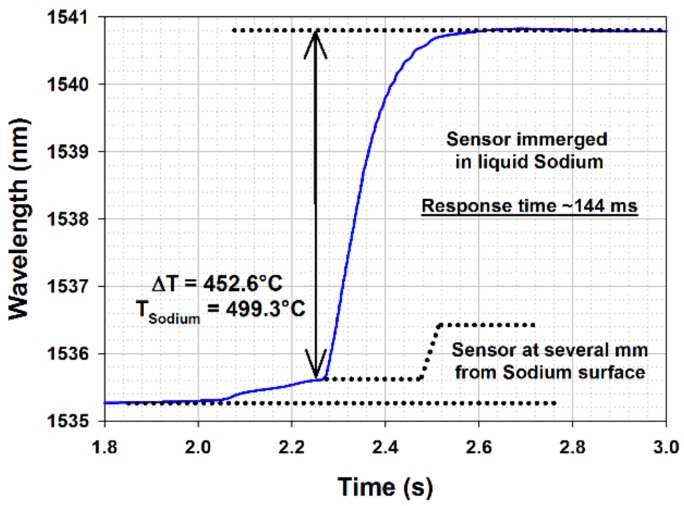
Bragg wavelength increasing during rapid immersion of the regenerated FBG-based temperature probe in sodium (T_Penelope_ = 46.7 °C; T_sodium_ = 499.3 °C).

With regard to thermocouples, the use of regenerated FBG for nuclear core on-line monitoring has considerable advantages not only in term of sensor multiplexing but also in term of response time with regard to temperature changes. With a monitoring system operating at a rate of 1 kHz (CEA system including a flattened ASE broadband source covering the C+L spectra window and an I-MON 512E spectrometer from Ibsen Photonics (Farum, Denmark), interfaced with an embedded computer), the response time of a FBG-based temperature sensor is limited by the thermal inertia of the sensing head itself: temperature changes have to diffuse through the metallic capillary itself (Inconel 600, outer diameter of 1 mm, inner diameter of 300 µm), through the residual air film between the optical fiber and the inner wall of the capillary, and through the polymer and silica claddings of the fiber itself. The FBG is located in the optical silica core which is roughly 10 µm in diameter. The response time of the regenerated FBG-based temperature probe has been tested through quick but manual immersion of the probe inside the liquid sodium heated up at different temperature. Therefore the measured response time of the probe is greater than the diffusion-limited value due to the delay induced by the manual immersion. Figure 14 shows a typical increase of the Bragg wavelength during immersion in sodium, with a change in temperature from 47 °C (ambient temperature inside the sealed Penelope facility) and 500 °C (temperature of the sodium inside the Sodium box shown on Figure 13 and heated thanks to a heating plate, the whole experiment being located and confined for safety reason within the sealed Penelope facility).

A slight increase in temperature is observed first when the probe is positioned close to the sodium surface. Then the probe is immersed in sodium: the observed rising time (10–90% step height) is equal to 144 ms. Repeated immersion experiments have been conducted according to different sodium temperatures.

**Figure 15:**
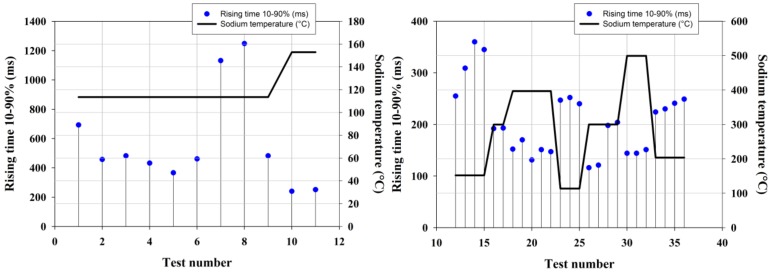
Analysis of regenerated FBG-based optical fiber temperature probe response time during immersion in liquid sodium heated at different temperatures.

Repeated experiments at low sodium temperature showed greater rising time as shown on Figure 15. The minimal observed value is between 300 ms and 700 ms, except two values above 1000 ms due to a too slow immersion. Reduced rising time is observed for sodium temperature increased up to 150 °C: the rising time then decreases down to around 250 ms. We thus expect these high rising time values to be related to a poor wetting of the outer metallic capillary wall by liquid sodium at low temperature. Similar experiments are conducted on a second regenerated FBG-based temperature probe packaged in an identical Inconel 600 capillary as shown on Figure 15 (right graph). For sodium temperature higher than 200 °C, the rising time is lower than 200 ms. Once the capillary has been immersed in sodium heated up to 400 °C, the rising time stays within 220–250 ms even at low liquid sodium temperature (below 200 °C). These results comfort the hypothesis that the high values observed at low temperature in our first experiment (left graph on Figure 15) may be imparted to a poor capillary wetting.

## 5. High Temperature Fiber Bragg Grating Strain Transducers

Besides their use as temperature sensor, regenerated and IR-fs-PbP FBGs could be used also as strain sensors able to operate in a high temperature regime. This is of utmost importance in order to deploy arrays of FBG transducers mixing temperature and strain sensors in order to propose an all optical fiber-based instrumentation for structural health monitoring (SHM) implementation in the nuclear industry. These high temperature resistant FBGs may be used not only in order to provide environmental data (temperature and strain) but also strain FBG sensors may be considered as acoustic receivers of elastic waves (such as Lamb waves) propagating into the structure under monitoring. This technology may propose a solution to realize continuous monitoring of nuclear power plants even during their operation. Usually performed during scheduled maintenance periods, continuous and even more SHM monitoring of nuclear reactors may lead to the advent of condition based maintenance of NPPs for specific components. Extreme FBG sensors may thus be a breakthrough in order to optimize their availability factor.

**Figure 16:**
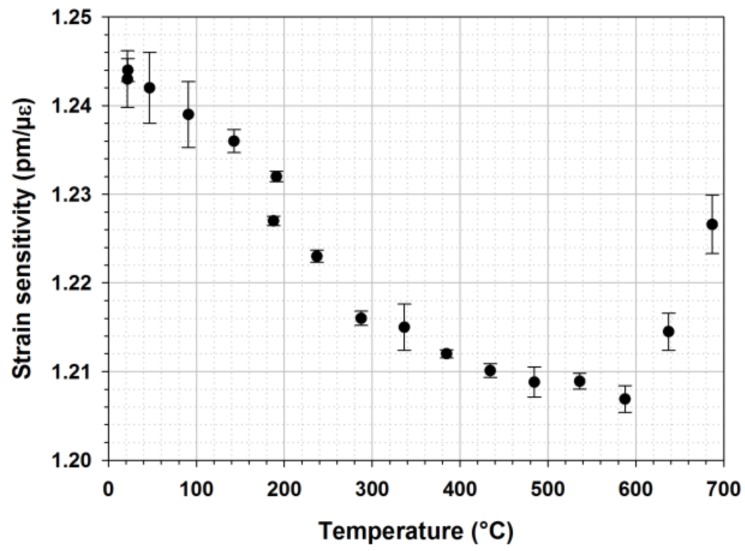
Strain sensitivity of a regenerated FBG for increasing operating temperatures up to 700 °C (~50 °C increments between two consecutive plots).

As a first step toward this ultimate goal, this study presents the calibration of regenerated FBGs versus strain at different operating temperatures. To do so a tensile test bed using a motorized translation stage with a 0.5 µm resolution has been used together with a split tubular furnace able to heat the grating up to 900 °C. A regenerated FBG is mounted into the setup. The experiment consists in pulling a 1 m long fiber containing the FBG from 0 to 3 mm with a 0.01 mm step, and return. The Bragg wavelength shift versus applied strain (up to 0.25%) is measured (using a tunable laser source T100S-HP and a CT-400 component tester, both from Yenista) and the strain sensitivity derived from these data is shown on Figure 16.

On Figure 16 one can clearly observe the decreasing strain sensitivity of this R-FBG with temperature up to 500 °C. Above 500 °C the strain sensitivity tends to increase: this may be attributed to a viscosity change of the silica material constituting the optical fiber. This behavior has to be taken into account when deploying such transducers for long term strain monitoring at high temperature: the strain sensitivity is not constant and change in silica thermodynamic properties (viscosity) has also an influence. Thus better control of the FBG is not sufficient: one has also to engineer the material itself to make the fiber sensing technology reliable. This could be achieved by drawing optical fibres with doped glasses presenting a higher vitreous transition temperature. Besides stabilizing the grating strain sensitivity with regards to the temperature, a critical issue for sensing application is also to find or develop efficient ways to attach the FBG strain sensors to any metallic structure in order to transduce efficiently the strain from the structure to the transducer itself, and this has to be done at high temperature. One approach to fix these FBG sensors to metallic substrate in order to perform strain measurement at high temperature is to bond these transducers using high temperature resistant adhesives. More sophisticated approaches may be to use metal-coated FBG and to braze them to the metallic structure or to directly embed the FBGs into a metallic substrate thanks to additive layer manufacturing processes [38,39].

## 6. Conclusions

Advanced manufacturing techniques using thermal engineering (regeneration process) and/or ultrafast laser micromachining of silica optical fibers have tackled the challenges of making high temperature resistant fiber Bragg grating sensors. High temperature resistant FBGs transducers are intensively studied for the monitoring of next generation nuclear facilities and especially for sodium-cooled fast reactors. Temperature mapping of the nuclear core is a major concern in order to prevent severe accidents due to coolant blockage along the fuel rod assemblies. In addition to temperature mapping, these FBGs are considered for strain measurements at high temperatures.

In this paper we have described two promising technologies for the realization of temperature resistant FBGs namely regenerated FBGs and IR-fs-PbP or “microvoids” femtosecond FBGs. R-FBGs are stable up to their regeneration temperature and preserve the spectral shape of a standard FBG at the expense of a reduced reflectivity. Before being deployed, R-FBGs are processed at high temperature to become thermally resistant: this so-called regeneration process reduces the mechanical reliability of the optical fibre. As shown in [26], a smf-28 optical fiber annealed at 900 °C —corresponding to the usual regeneration temperature for a seed FBG written in H_2_-loaded smf-28 fiber–shows a breaking stress of 0.1 GPa with regards to 4.5 GPa for the pristine fibre. Besides decreasing the regeneration temperature that may also leads to a decrease of the operational thermal range of the regenerated grating, two main solutions can be considered to overcome this issue: (i) to perform the regeneration at the initial step of the process to monitor subsequently (in situ regeneration) provided the available temperature is high enough, or (ii) to minimize the mechanical impact of the regeneration at 900 °C to the optical fiber as detailed in [26] by thermally processing only the fiber section containing the seed FBG (e.g., by using compact furnace or CO_2_ laser-based regeneration setup). IR-fs-PbP FBGs have a thermal stability beyond 1000 °C and may be photowritten without removing the protective (polyimide) coating of the optical fiber, thus optimizing the mechanical reliability of the sensor as demonstrated in [8] but for type II IR femtosecond FBGs (not IR-fs-PbP grating) inscribed using a phase mask through the fiber’s polyimide coating and showing a breaking stress of 0.5 GPa but without high temperature treatment prior to the pull-test. More detailed studies on the mechanical reliability of all kind of high temperature resistant FBGs is mandatory to assess clearly their limit especially in anticipation of strain measurements at high temperature.

Both R-FBGs and IR-fs-PbP FBGs are studied for thermal and strain measurement at high temperatures. Regenerated and IR-fs-PbP FBGs are relevant fiber optic transducers in order to develop on-line monitoring instruments and to implement structural health monitoring systems into existing nuclear power plants and into the next generation of nuclear reactors.

## Figures and Tables

**Table 1:** Radiation-induced error on temperature measurements made with a regenerated FBG.

Temperature (°C)	Bragg Wavelength Sensitivity to Temperature (pm/°C) (Extracted from [3])	Temperature Measurement Error of A Regenerated-FBG After Receiving A 33 Kgy Dose
Room temperature	9.5	4.0 °C
200 °C	12.7	2.5 °C
400 °C	14.6	1.3 °C
600 °C	15.7	0.3 °C
800 °C	16.4	0.3 °C

**Table 2:** Spectral characteristics measured before and after the long term thermal annealing at four distinct temperatures of four IR-fs-PbP FBGs written in SMT-A1310H optical fibre. ΔR/R and Δλ_Bragg_ stand respectively for the normalized change of the Bragg peak maximum amplitude and of the Bragg wavelength shift before and after annealing, at room temperature.

λ_Bragg_start_ (nm)	R_Start (%)	T_Annealing (°C)	Δλ_Bragg_ (nm)	ΔR/R (%)
**1530**	12%	950 °C	−0.52 nm	−43%
**1540**	16%	945 °C	−0.43 nm	−5%
**1560**	25%	940 °C	−0.41 nm	−39%
**1570**	8%	925 °C	−0.18 nm	+5%
